# Assessing shade matching capability of Omnichroma, a single shade composite in posterior restorations: an in vitro study

**DOI:** 10.25122/jml-2024-0210

**Published:** 2024-08

**Authors:** Sana Baghizadeh, Kasra Tabari, Kamyar Abbasi, Seyedeh Farnaz Tabatabaei, Haleh Heshmat

**Affiliations:** 1 Private Office, Tehran, Iran; 2 Department of Operative Dentistry, Shahid Beheshti University of Medical Sciences, Tehran, Iran; 3 Department of Prosthodontics, School of Dentistry, Shahid Beheshti University of Medical Sciences, Tehran, Iran; 4 Operative Dentistry Department, Faculty of Dentistry, Tehran Medical Sciences, Islamic Azad University, Tehran, Iran

**Keywords:** resin composite, blending, color adjustment potential, shade match, dentistry, conservative dentistry

## Abstract

Recent composites are being developed to simplify shade matching in composite restorations. Only a limited amount of research has been conducted to determine the optical performance of this newly introduced composite in this area. This study investigated the Omnichroma (OMN) color matching (a single shade composite within type-I restorations) via simulated clinical cavities. A total of 72 frames were created by occupying the mold with Estelite Σ Quick (ES) of A1, A2, and A3 shades (*n* = 24). Each shade of composite frame was divided into three subgroups (*n* = 8) according to cavity dimension (width = 2, 3, and 4 mm/depth = 2 mm). Cavities were filled with Omnichroma. Color parameters were calculated based on CIEDE2000 (ΔE00) using a non-contact spectrophotometer. Finally, the data were analyzed using a two-way ANOVA (the Tukey HSD test) (*P* = 0.05). The surrounding frame color significantly affected the color-matching capacity of OMN (*P* < 0.0001). Groups A1 and A3 showed the lowest and highest amounts of ΔE00, respectively. The cavity width also influenced the color-matching ability of OMN (*P* < 0.0001) significantly. According to the results, 4 mm cavity width showed the lowest amount of ΔE00, and 2 mm showed the highest amount. Monochromatic composites (OMN) did not match colors well in Class I cavities in posterior teeth. In cases of teeth with less chromatic surroundings, OMN matched shades better. OMN could better match shades in posterior teeth with wider cavities.

## INTRODUCTION

Composite resins are widely used as restorative materials for all teeth due to their advanced mechanical, physical, and aesthetic properties [1-3]. Composite resins are commercially available in several enamel and dentin shades with varying translucency and opacity as measured by the VITA classic shade guide. In this regard, matching the resin composite color with the restored encircling teeth is challenging [4-7]. Thus, meticulous shade-matching strategies are needed, including shade selection by clinicians or tools and 'stratification', also known as the 'natural layering concept', using composite resins of different shades and opacities. Since 1980, resin composite restorations have been layered [8-10]. This procedure includes applying resin composites with various opacities and chromas for each layer to simulate the optical features of natural teeth. Although the layering technique has shown satisfactory results, it is much more complicated than the conventional two- or one-shade procedure. It requires additional technical qualities and clinical chair time [11-14]. The color-associated features of composite resins are closely related to their esthetic appearance, explaining why clinicians seek materials that mimic natural dentition [15,16].

Composite resins that show color blending with the encircling tooth being restored may also have a clinical benefit [1,17,18]. This procedure may simplify shade selection, enhance the esthetic look of the restoration, lessen the number of composite resin shades needed, and compensate for shade selection errors to some degree [17-19]. Recently, Omnichroma (OMN) (Tokuyama Dental, Tokyo, Japan), a new advanced single-shade composite resin, has been developed to lessen the dependence of final restoration esthetic on the shade-matching skills of clinicians and reduce the chair-side time needed for each patient. The producer claims that the structural shade of OMN adapts to the color of the encircling tooth, regardless of its shade [20]. The mentioned structural color is precipitated by incorporating spherical homogenized filler particles with a size of 260 nm through the 'sol-gel technique'. This technique is applied to provide uniform filler particles, leading to the color adjustment potential of OMN [21].

Light illumination via the resin composite spreads across the filler particles’ surface in different directions. Light transmission through composite resin is composed of direct transmission and diffusion. Resin composite filler particles can affect the light transmission properties. In addition, the blending effect of a restoration can be affected by the refraction propagation and scattering of light via the resin composite [22]. As a novel technological innovation, the filler morphology is used for shade matching, where emission of the resin composite and light absorption are performed for shade matching. This feature is called 'structural color' [23]. Structural colors are specifically caused by the physical interaction of super-nano periodic structures of substances using light through refraction and reflection [24-26]. Super-nano-filled resin composites, which provide shading primarily through structural color, offer potential advantages such as reduced shade change and color distortion over time due to decreased photochemical degradation. The explanation is that the arrangement of their filler particles matches visible wavelengths [27-29]. The purpose of this research was to investigate the color matching of OMN in Class I restorations using simulated clinical cavities as a means of determining the color.

## MATERIAL AND METHODS

### Simulated cavity preparation

**Figure 1:**
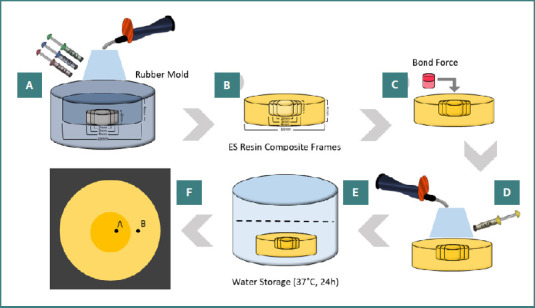
The schematic representation of specimen preparation for color measurement. A, ESQ resin composite frames made from cured silicone impression substance; B, ESQ resin composite frame with simulated Class I cavity; C, adhesive application; D, the cavities were occupied with OMN and polymerized; E, after polishing the surface of the restorations, they were kept for 24 hours in 37^o^ water; F, measuring the simulated restoration and the surrounding resin composite frame.

The institutional research committee (IR.IAU.DENTAL.REC.1401.034) submitted this paper. Table 1 presents the details of the tested materials in this work. A customized disk-shaped silicon mold (diameter = 10 mm and height = 4 mm) was made using a clear additional silicone impression material (Kristal, Müller-Omicron GmbH & Co. KG) as shown in Figure 1A [30]. In this research, 72 frames were built by occupying the mold with Estelite Σ Quick (ES) (Tokuyama Dental) of A1, A2, and A3 shades (*n* = 24). Each shade of composite frame was divided into three subgroups (*n* = 8) according to cavity width. Simulated Class I cavities were prepared with three widths (2, 3, and 4 mm), as shown in Figure 1B. The cavity depth was constantly 2 mm in all tested groups. Preparation was done with a coarse grit 007 diamond bur (Drendel + Zweiling, kalletal) in a contra-angle handpiece (NSK). The bur was discarded after every five preparations. A clear index (Kristal, Muller-Omicron GmbH & Co.KG) was made from the first approved preparation to ensure the standardization of the cavities. In all simulated cavities, at first, the Palfique bond (Tokuyama Dental) was used based on the instructions of the manufacturer and polymerized using a light curing unit (Bluephase G2, Ivoclar Vivadent, Amherst) for 20 s (Figure 1C). The irradiance was 1300 mW/cm^2^, confirmed after each replication through a radiometer (Bluephase Meter 2, Ivoclar Vivadent, Amherst) (Figure 1D) [31, 32].

**Table 1:** Materials and their main components

Material (Manufacturer)	Composition	Filler type (batch number)	Shades
Omnichroma (Tokuyama Dental)	Fillers: round-shaped composite filler (containing 260 nm spherical SiO2-ZrO2), 79 wt% uniform sized supra-nano spherical filler (SiO2-ZrO2 260 nm), Base resin: UDMA, TEGDMA	Nanofilled (17J23)	-
Estelie Σ Quick (Tokuyama Dental)	Fillers: 82 wt% uniform sized supra-nano spherical filler (SiO2-ZrO2, SiO2-TiO2 100-300 nm (average 200 nm)), round-shaped composite filler (100–300 nm (average 200 nm), spherical SiO2-ZrO2) Base resin: Bis-GMA, TEGDMA	Nanofilled (J0662)	A1, A2, and A3

The simulated type-I cavities were then occupied with Omnichroma composite resin (OMN) (Tokuyama Dental) in a single increment. The stamping technique was used to ensure that the final restoration matched the initial structure of the disc. In this method, a transparent occlusal stamp was made from the surface of the unprepared ES composite disc using the mentioned transparent additive silicone. After placing the OMN composite in the cavity, the stamp was pressed. Next, after removing the flashes of the composite, it was polymerized with the light curing unit as described above. Therefore, the tip of the light cure device was placed precisely adjacent to the composite. Ultimately, the samples’ surface was covered with a glycerin layer (Oxybar, Cobalt-Biomed) of 5 mm thickness and post-cured for an extra 20 s to diminish the oxygen-inhibited layer. The glycerin was washed off the samples for 20 s [33, 34].

Subsequently, restorations were polished with a one-step polisher point for 40 s, PoGo (Dentsply, York), using light hand pressure in a circular motion. Afterward, the polymerized composite restorations were kept in distilled water for 24 hours at 37 C (Figure 1E) [35].

### Shade matching evaluation

Color parameters were measured for each simulated composite restoration and the composite resin frame utilizing an illuminance spectroradiometer (cs-2000, Konica Minolta) (Figure 1F). The measurements were performed using two fiber optic cables (model 70050, Newport Strat-ford Inc. Franklin) and a xenon lamp (300W, Newport Stratford, Franklin). Reflection values of a 1-mm-circular area in the center of each composite sample were transformed into CIELAB color parameters (D65 illuminant, 2^o^ standard observer). The spectrophotometer was calibrated using a white calibration plate (cs-A5) under controlled illumination [36, 37].

### Statistical analysis

According to the results of the two-way analysis of variance (ANOVA) power analysis option of PASS II software (α = 0.05 and β = 0.1), the average standard deviation of ΔE was equal to 0.85, and the effect size was 0.58. The sample size needed for each study group was 8 [36]. A two-way ANOVA was conducted to compare the effects of cavity dimensions and surrounding shades on color difference (ΔE00). Tukey’s Honestly Significant Difference (HSD) test was used for post-hoc comparisons to evaluate the impact of each factor on the amount of ΔE00.

## RESULTS

The results revealed that the interaction effect of the width and surrounding shade of the cavity was not significant (*P* = 0.08). Table 2 represents mean color differences (ΔE00) and SD values.

**Table 2:** The mean color differences (ΔE00) and SD for simulated cavities

Frame shade	Cavity width (mm)	ΔE00 (SD)
A1	2	3.39 (0.38)
	3	1.79 (0.53)
	4	0.88 (0.44)
A2	2	3.83 (0.42)
	3	2.50 (0.67)
	4	1.93 (0.41)
A3	2	4.08 (0.53)
	3	2.31 (0.63)
	4	3.27 (0.64)

The effect of surrounding shade on ΔE00 was statistically significant in all groups (*P* < 0.0001). The average ΔE00 in A1 Group (2.02) was significantly lower than in Groups A2 (2.75) and A3 (3.27) (*P* < 0.0001). The average ΔE00 in Group A2 was also considerably lower than in Group A3 (*P* = 0.004).

The effect of cavity width on ΔE00 was significant in all groups (*P* < 0.0001). Among all groups, specimens with a cavity width of 4 mm had significantly lower ΔE00 values (1.71), while specimens with a cavity width of 3 mm (2.57) and 2 mm (3.77) had significantly higher values (*P* < 0.0001). Similarly, the mean ΔE00 in the specimen with a cavity width of 3 mm was significantly lower than the mean ΔE00 in the specimens with a cavity width of 2 mm (*P* < 0.0001).

**Figure 2:**
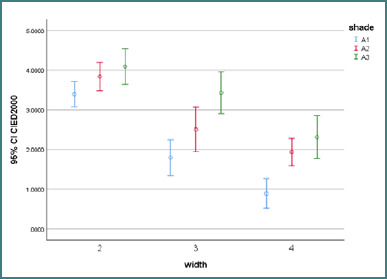
ΔE ± 95% CI values for CIEDE2000 analysis between various shades for each group

The effect of surrounding shade and cavity width on the amount of ΔE is summarized in Figure 2.

Two-way ANOVA was performed to compare the effects of width and surrounding shade on the lightness difference (ΔL00). The results revealed the nonsignificant interaction effect of the cavity’s width and surrounding shade (*P* = 0.2). Tukey’s HSD test was applied to compare the effect of the abovementioned factors on ΔL00. Mean color differences (ΔL00) and SD values are presented in Table 3.

**Table 3:** Mean ΔL00 and SD for simulated cavities

Frame shade	Cavity width (mm)	ΔL00 (SD)
A1	2	-5.12 (2.83)
	3	-4.34 (1.93)
	4	-3.99 (2.40)
A2	2	0.71 (1.48)
	3	1.01 (1.71)
	4	-0.48 (2.35)
A3	2	2.32 (2.58)
	3	0.13 (2.90)
	4	1.27(2.45)

**Figure 3:**
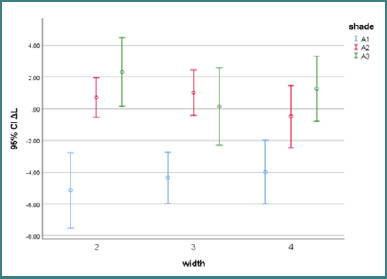
ΔL ± 95% CI values for CIEDE2000 analysis between various shades for each group

The effect of surrounding shade on the average ΔL00 was significant in all groups (*P* < 0.0001). The average ΔL00 in the A1 Group (-4.84) was significantly lower than in the A2 (0.14) and A3 (1.24) groups (*P* < 0.0001). No statistically significant difference was found between Groups A2 and A3 (*P* = 0.44). The effect of cavity width on the average ΔL00 was not significant in any group (*P* = 0.8). The average ΔL00 was reported as -1.06, -1.06, and -0.69 in specimens with cavity widths of 4, 3, and 2 mm, respectively. The effect of surrounding shade and cavity width on the amount of ΔE is summarized in Figure 3.

## DISCUSSION

The current in vitro work revealed that the shade-matching ability of OMN with the encircling specimen increases in lighter surrounding shades and wider cavities. The manufacturer describes OMN as a one-shade resin composite without pigments that matches all VITA classical shades A1-D4. The composition and characteristics of the material influence the color properties. These color characteristics are obtained using a supranano spherical filler made of uniform 260-nm particles of silicon dioxide (SiO2) and zirconium dioxide (ZrO2). After polymerization, the monomer is precisely the right shape and size to produce a color ranging from red to yellow without additional dyes or pigments. This red-to-yellow structural color blends with the nearby tooth color and the reflected light in an additive color mixing procedure, maximizing OMN’s ability to tone with natural teeth [34,37,38]. Uniform SN filler particles in a specific size range can exhibit vibrant structural colors due to the Bragg diffraction of white light caused by the face-centered cubic structure of silica photonic crystals [30]. The resin matrix influences the composite resin’s transparency, as composites comprising Bis-GMA are more luminous than those without. The translucency is positively related to the blending effect [33]. This result is consistent with those of a previous study revealing a connection between the blending effect and translucency that reflected the surrounding dental tissues. The material becomes more translucent after polymerization because the refractive index of the monomers changes. It has been used to prepare resin composites that reflect a definite wavelength within the tooth color space and respond to light waves at a specific frequency [31,37].

As a rule, human tooth shades fall within a limited range of red-orange-yellow tones. Therefore, a single-shaded resin composite would match the A shades of VITA classic in almost all cases, as more than 1/2 of the natural teeth shades fall into the orange range [30]. Also, A1, A2, and A3 are the most common VITA shades for anterior teeth [39]. Therefore, in this study, a classic shade of VITA was investigated. Shade matching has been assessed in vitro using simulated tooth preparations instead of real human teeth by many researchers [23,30]. Natural human tooth color is caused by complicated interactions between tooth and light, which is affected by factors like tooth type, position, and age [40]. Therefore, rather than using natural teeth initially, a simplified simulated clinical cavity model can be used to determine the shade match of an esthetic resin composite in a laboratory setting before starting a more expensive clinical procedure. The ability of three resin composites to match colors at the composite-tooth interface was examined by Mourouzis *et al*. However, they found no statistically significant differences between them. Visually, the distinctions were also minimal. They concluded that the restorations might be non-differentiable clinically in such situations due to the polychromatism and translucency of the teeth next to them. As in the present study, these observations support using resin composite discs in vitro to predict clinical shade matching [41]. In this research, a spectroradiometer was used to assess shade-matching capability on genuine-size cavities. While a contact spectrophotometer is accurate, a spectroradiometer performs more precisely [42]. In this research, the CIEDE2000 color difference formula was employed to investigate how resin composites' translucency and color shift towards the surrounding material. This formula, based on the CIELAB color space, was recommended by the International Commission on Illumination (CIE). Although CIELAB is known for its acceptable color quantification capabilities, the CIEDE2000 is significantly more sophisticated and aligns better with visual perception. Despite its complex computational method, CIEDE2000 was recommended for minimal viewing conditions due to its superior accuracy. Practically speaking, this equation has been utilized under various circumstances, especially in recent research in dentistry [30, 31].

In this study, OMN matched the specimen’s lighter colors more accurately. Previous research demonstrated that the color of the cavity floor could affect the color appearance of resin composites, which in turn is greatly affected by the resin composites’ translucency [32]. Iyer *et al*. [33] demonstrated that OMN visually matched lighter shades better. They found that the function of single-shade composite systems could not be predicted by increasing the acrylic teeth chroma and decreasing their value. Their findings are consistent with what we found in our study, as OMN blended better with lighter shades like A1. Pereira *et al*. [23] investigated the ability of OMN to mix with the 16 VITA classical A1-D4 shades in simulated Class-I cavities made in denture teeth. They concluded that OMN had the smallest ∆E values compared to other composite materials. However, the opposite conclusion might be reached when composite frames instead of acrylic teeth are used. No study has been performed on the effects of cavity width on the shade-matching capability of OMN. However, we found a positive correlation. Previous studies showed that cavity dimensions might influence the blending effect due to alterations in the optical features of the remaining tooth structures, with smaller cavities displaying a better blending effect [10]. Other studies demonstrated that the sample thickness significantly influenced the optical performance of the resin-based composites that were assessed. The transmittance decreased by increasing the thickness, while the scattering and absorbance increased [35]. The impact of restoration depth on color-matching ability was examined by Akgül *et al*. They found that an increase in cavity size enhances the blending effect of OMN due to variations in the tooth structures’ optical behavior. Regardless of the restoration size, increasing the restoration thickness allows the substances used to reflect the color properties of the tooth better. A previous study assessed the impacts of restoration depth on the color-matching ability of the same restorative material. It was concluded that 3.0-mm restorations had higher color-matching ability values than 2.0-mm ones [34].

Poggio *et al*. evaluated four flowable resin composites after exposure to acidic and alcoholic beverages. A Leica DCM 3D microscope was used to examine each specimen: the arithmetical mean height of the surface profiles was measured (Sa). There were higher Sa values in Coca-Cola groups. Regarding surface texture, there were no significant differences between the specimens of the different materials. Neither control conditions nor the Cola application showed significant differences among TetricEvoFlow, Esthet-X Flow, and Amaris Flow. In control and Coca-Cola-treated conditions, the Sa values of SureFil SDR flow were significantly higher. According to their findings, all flowable resin composites evaluated eroded their surface roughness when exposed to acidic and alcoholic drinks [43].

CIEDE2000 was used by Abdulmajeed *et al*. [44] to determine wear and color stability for high-viscosity bulk-fill and conventional resin composites, both with and without PH. To evaluate wear, 32 disc-shaped specimens were prepared with Filtek One Bulk Fill Restorative (FOBFR; 3M ESPE) and Filtek Supreme Ultra (FSU, 3M ESPE). PH and RT groups showed greater wear for FSU than FOBFR. The materials were subjected to increased wear due to PH. After aging, there was a significant color change regardless of time or resin composite type. Resin composites were color stable regardless of PH. The high-viscosity bulk-fill composite demonstrated superior wear resistance compared to conventional resin composites. In addition to increasing its wear susceptibility, PH resin composites do not affect their color stability [44].

To determine possible differences in adhesion of different restorative materials to *Streptococcus mutans* strain (CCUG35176), Poggio *et al*. investigated the adhesion of different restorative materials. They used colony-forming unit (CFU) determination, where bacterial suspensions were deposited onto each material to evaluate adhesion. Compared to posterior packable composites P60 and glass ionomers, silorane-based composites were less adhesive. Fluoride had no effect on the attachment of *S. mutans* to glass ionomers. Surface chemistry and electrostatic forces between bacteria and restorative surfaces can contribute to differences in bacterial adhesion [45]. The new supra-nano-filled esthetic resin composite was examined in this investigation, being the first research study using this method in esthetic dentistry. However, the benefits of structural color technology are applied extensively in several fields, including cosmetics, display technologies, and textile engineering. This study used simulated Class I cavities, which was one of the limitations. The OMN color parameters in natural teeth, both in vitro and in vivo, require additional research. Furthermore, comparing the results with those obtained using more recent universal resin composites is necessary.

## CONCLUSION

Within the limitations of the present study using disk-shaped specimens, it can be concluded that the monochromatic composite (OMN) did not exhibit satisfactory color-matching ability in Class I cavities in posterior teeth. However, the shade-matching ability of OMN improved when the surrounding tooth shade was less chromatic, and the cavities in the posterior teeth were wider.

## Data Availability

Further data is available from the corresponding author upon reasonable request.

## Personal thanks

This study was carried out as a student thesis to receive a doctorate in dentistry. The authors would like to thank Dr. Mohammad Javad Kharazifard (Tehran University of Medical Sciences) and Dr. Razieh Jafari (Institute for Color, Science and Technology) for their assistance.
